# Inability to Reverse Aspirin and Clopidogrel-induced Platelet Dysfunction with Platelet Infusion

**DOI:** 10.7759/cureus.3889

**Published:** 2019-01-15

**Authors:** Stephen M Cohn, Jean-Carlos Jimenez, Leen Khoury, Javier Martin Perez, Melissa Panzo

**Affiliations:** 1 Surgery, Staten Island University Hospital, Staten Island, USA; 2 Surgery, Hackensack Meridian Health, Hackensack, USA; 3 Emergency Medicine, Staten Island University Hospital, Staten Island, USA

**Keywords:** platelet dysfunction, aspirin and clopidogrel effects, bleeding time, platelet transfusion, storage defect

## Abstract

Background

Platelets are commonly administered to trauma patients to reverse the effects of pre-injury anti-platelet drugs if these individuals are judged to be at risk for ongoing bleeding (i.e., traumatic brain injury). In the U.S. blood banks, platelets are maintained at room temperature and are not infused prior to 72 hours storage due to rigorous screening methods. Recent work suggested that cold refrigerated platelets may be effective at restoring platelet function. We hypothesized that refrigerated platelets might be superior to room temperature platelets in reversing aspirin and clopidogrel-induced platelet dysfunction.

Methods

Using a cross-over design, 10 healthy, adult subjects underwent platelet removal by apheresis, received anti-platelet drugs (aspirin 325 mg and clopidogrel 75 mg) daily for three days, and then had return of their own platelets (about 3 x 10^11 ^platelets). Five subjects were randomly assigned to receive platelets stored at 4°C, and five received platelets stored at room temperature. One month later, this entire process was repeated with each subject receiving platelets stored by the alternative method. Thus, subjects served as their own controls. At multiple time points during the study in vivo platelet function was assessed by bleeding times, which were measured by a single observer blinded to patient group.

Results

Bleeding times rose dramatically after anti-platelet drugs were given, but remained well above the normal range (seven minutes) despite reinfusion of platelets. There were no differences in platelet function according to the method of storage.

Conclusions

Transfusion with autologous platelets appears to be ineffective in reversing the anti-platelet effects of aspirin and clopidogrel. Cold refrigerated platelets were no more effective than room temperature stored platelets in restoring platelet function.

This abstract was presented at American College of Surgeons-clinical congress, Boston 10-22-2018.

(Khoury L, Cohn S, Panzo M. Inability to Reverse Aspirin and Clopidogrel-Induced Platelet Dysfunction with Platelet Infusion. Journal of the American College of Surgeons. 2018. 227. S265. DOI: 10.1016/j.jamcollsurg.2018.07.546).

## Introduction

Over the last few decades, aspirin and clopidogrel have emerged as important pharmacological therapy to decrease the incidence of thrombosis in patients who are at high risk for cardiovascular occlusion [1]. The drugs work by selectively and irreversibly inhibiting mediators responsible for platelet aggregation, and have been shown by some [2] to reduce restenosis rates when used in patients who have undergone cardiovascular stenting or revascularization procedures. However, these patients have an increased risk of bleeding complications [3]. This tendency to bleed represents a significant potential problem as over 100 million patients have been prescribed clopidogrel in the last decade [4].

Hemorrhage following injury may be particularly problematic in patients receiving anti-platelet therapy. Trauma patients often require life-saving interventions related to their injuries and drug-induced platelet functional impairment may exacerbate surgical bleeding and increase morbidity. The current standard management, (infusion of platelets), may be suboptimal in reversing the anti-platelet effects of aspirin and clopidogrel. Previous primate studies demonstrated that platelet transfusion was ineffective in reversing anti-platelet drug effects when the platelets infused were older than 48 hours when stored at room temperature [5]. Platelets given in the United States are typically stored at room temperature and must remain in the blood bank for more than 48 hours secondary to screening tests performed on the donated sample to avoid infectious disease transmission. Alternatively, the use of platelets stored at 4°C for less than 72 hours is postulated to improve reversal of the anti-platelet effect caused by the drugs, and was recently approved by the Food and Drug Administration (FDA) [6].

We investigated the impact of platelet storage temperature on platelet function and the ability of platelet infusion to reverse the effects of anti-platelet drugs. We hypothesized that refrigerated platelets were superior to room temperature platelets in reversing aspirin and clopidogrel-induced platelet dysfunction.

## Materials and methods

Trial design

This was a randomized, single-blinded, crossover pilot study conducted at Staten Island University Hospital, Staten Island, New York. The Institutional Review Board of Northwell Health/Feinstein Institute provided protocol approval. Written informed consent was obtained from all participants.

Trial procedures

Ten healthy adult subjects participated and had not received aspirin or clopidogrel (or any other anti-platelet medication) in the previous 30 days. After platelet donation, participants received anti-platelet drugs (aspirin 325 mg and clopidogrel 75 mg) daily for three days, and then received return of their own platelets (stored at 4°C or room temperature). In this cross-over trial, subjects were randomly assigned to receive platelets stored at 4°C or room temperature. One month later, this entire process was repeated with each subject receiving platelets stored by the alternative method. At fixed time points (days one, three, and seven post-infusion) skin temperature and bleeding time (BT) were measured.

Bleeding Time Technique

In order to reduce variability, only one research staff member performed each of the bleeding time measurements. We used the Surgicutt® Method (according to the manufacturer recommendations provided in the package insert), a modified Ivy bleeding test that allows for improved accuracy. This method uses a Surgicutt® Adult device (International Technidyne Corporation, Edison, NJ) that has a trigger and spring method for the blade. The blade has a depth of 1.0 mm and a width of 5.0 mm, which eliminates variability of blade precision. To prepare the subject for the bleeding time test, the subject was seated with their elbow slightly flexed and the forearm resting on a sturdy surface. A sphygmomanometer cuff was placed over the arm above the antecubital fossa. The forearm was cleansed with an alcohol prep wipe and allowed to air dry. Once dry, triplicate measurements of surface skin temperature were made using a Klein Tools® IR 1000 Infrared Thermometer (Klein Tools, Inc, Lincolnshire, IL) over the volar surface of the forearm. The area being measured was 5 cm (three finger widths) distal to the antecubital fossa. At this point, the cuff was inflated and maintained at 40 mm Hg. After one minute, the Surgicutt template was gently placed parallel to the antecubital crease so all four sides make contact with the skin and create a uniform incision. Care was taken to avoid any superficial veins. Then the trigger is pressed and a stopwatch was started simultaneously. After 30 seconds from incision, WhatmanTM Grade GB003 filter paper (GE Healthcare, Sanford, ME) was used to blot the blood while staying away from the incision site so as not to disturb the clot formation of the wound. This was performed at 30-second intervals using an unused side of the filter paper until there was no more blood staining of the filter paper. Once the bleeding stopped, the stopwatch was stopped and the blood pressure cuff was deflated and removed. The bleeding time was recorded to the nearest 30 seconds. Any bleeding time test that lasted more than 20 minutes (20 minutes and 30 seconds) was also stopped and recorded as >20 minutes.

After the baseline bleeding time, the subject underwent platelet apheresis. The subject then received aspirin 325 mg and clopidogrel 75 mg daily for a total of three doses (the first dose was given 20 minutes after platelet donation). During the following two days, the investigators communicated with subjects via phone calls to confirm the time of medication administration. The subjects returned 72 hours later (day four) underwent pre-transfusion bleeding time to confirm the anti-platelet drug effect, and then received the first autologous platelet transfusion (3 x 10^11^ platelets were infused). The research staff member performing the bleeding times was blinded as to the platelet storage group assignment (which was randomly allocated) and was not present during transfusion. Twenty minutes after transfusion, a post-transfusion bleeding time was performed to determine the efficacy of platelet transfusion. Post-transfusion bleeding times were also measured at days one, three, and seven after platelet transfusion.

Platelet Storage

All platelets that were randomized to the Room Temperature Sequence were stored according to the Standard Operating Procedure at our center (Staten Island University Hospital, Donor Center Policies and Procedures, September 1, 2015, Manual Code D-25.0). When single donor platelet (SDP) products are collected and placed in storage on the platelet rotator, the room temperature is measured and recorded from a thermometer in the immediate vicinity of the rotator. This measurement was recorded every four hours and the temperature maintained between 20 and 24°C.

Platelets randomized to the Refrigerated Sequence were collected and placed in the donor center refrigerator. Refrigerator temperatures were recorded every four hours on control log and maintained between 1 and 6°C.

The research staff member that was responsible for the bleeding times remained blinded to the randomization sequence. We repeated this same protocol four weeks after initial donation when each of the participants received platelets stored by the alternate method. The primary outcome was bleeding time at one-hour post platelet transfusion.

It should be noted that in vitro assessments using the VerifyNow system were planned to accompany the in vivo Bleeding Time studies, but we were unable to perform the measurements due to the unavailability of the machine.

Statistical analysis

This was a pilot study, power calculation was not performed. Only 10 patients were needed to do the pilot study. We used a paired t-test (or Wilcoxon Signed-Rank test when appropriate) to evaluate the impact of anti-platelet drugs on bleeding time. We compared each subject, the change from baseline to follow-up visits with methods of platelet storage.

Descriptive results are presented for changes from baseline in bleeding time for the two groups over time. We used a mixed-effect model with treatment sequence, treatment period, treatment, and treatment-by-period interaction as fixed effects and subject nested in sequence as a random effect. If the treatment‐by-period interaction effect was found to be statistically significant, we analyzed the subject comparison to the first time period only by using an analysis of covariance model. The same analysis was done at each time point but the analysis at one-hour post platelet transfusion was the primary analysis.

## Results

Ten healthy volunteers underwent platelet removal by apheresis, received anti-platelet drugs for three days, and then received return of their own platelets (stored at 4°C platelets or room temperature platelets). During screening visits, the blood bank measured subjects weight mean of 78.82 kg (range 62.6 to 95.3) and performed a blood draw for complete blood count testing (a standard blood bank protocol prior to any elective platelet donation). On visit number one, hemoglobin (mean hemoglobin 14.81 g/dL ± 0.9), hematocrit (mean hematocrit 44.08% ± 2.4), platelet count (mean 264.1 uL ± 37.1) and platelet volume (mean 9.99 fL ± 1.1) were assessed. On visit number two values were comparable to the pretransfusion evaluation: hemoglobin (mean 14.75 g/dL ± 0.8), hematocrit (mean 43.9% ± 1.5), platelet count (mean 259 uL ± 39.6) and platelet volume (mean 9.89 fL ± 0.9) (Table 1).

**Table 1:** Screening visits.

Visit #1					
Subject	Hemoglobin (g/dL)	Hematocrit (%)	Platelet Count (uL)	Mean Platelet Volume (fL)	Pre-infusion Platelet Volume (mL)
001	15.3	45.9	282	9.1	242
002	12.8	39.3	300	10.1	236
003	15.1	45.0	212	11.1	244
004	14.8	46.0	267	12.1	238
005	15.9	45.9	246	11.0	238
006	15.6	45.8	266	9.8	246
007	13.7	41.1	247	9.4	246
008	15.6	46.0	213	9.6	240
009	15.2	43.3	333	9.3	244
010	14.1	42.5	275	8.4	246

Visit #2					
Subject	Hemoglobin (g/dL)	Hematocrit (%)	Platelet Count (uL)	Mean Platelet Volume (fL)	Pre-infusion Platelet Volume (mL)
001	14.7	43.1	275	9.2	242
002	13.3	41.5	331	9.8	244
003	15.4	45.0	210	10.7	238
004	15.4	46.4	293	11.2	238
005	16.3	45.8	274	11.2	246
006	14.7	43.2	258	9.4	242
007	14.3	42.9	257	9.6	230
008	15.0	45.1	190	10.1	242
009	13.8	43.6	261	9.2	238
010	14.6	43.1	246	8.5	234

The subjects were randomized to receive room temperature platelets on first visit then 4˚C platelets on the second and vice versa. Bleeding time was measured before starting anti-platelets drugs and documented as baseline bleeding time with a mean of 4.75 min, median 4.5, and standard deviation of 0.857 in the 4˚C platelets treatment arm and mean of 4.45 min, median 4, and standard deviation of 1.64 in the 22˚C platelets treatment arm. Skin temperature was assessed prior bleeding time measurement (mean 88.98˚F in the 4˚C platelets treatment arm versus 89.77˚F in the room temperature treatment arm). The healthy volunteers started the anti-platelets drugs and after three days returned to our blood bank where we performed pre-transfusion skin temperature measurement and bleeding time; bleeding time rose dramatically after anti-platelet drugs were given with a mean of 15.9 min, median 20.5, and standard deviation of 6.2 with normal skin temperature measurements (mean 88.68˚F) in the 4˚C platelets group and mean of 14.60 min, median 14.75, and standard deviation of 5.961 with normal skin temperatures measurements (mean 89.05˚F) in the 22˚C platelets group (p < 0.05).

We measured bleeding time after one hour, one day, three days, and seven days post-transfusion; mean bleeding times were 12.7, 12.4, 9.5, and 6.3 min; median 12, 10.5, 7.5, and 5.5; and standard deviation of 5.4, 5.9, 5.6, and 3.6, respectively, for 4˚C platelets. Skin temperature was measured before bleeding time in each time point, means were 88.56, 88.57, 88.43, and 87.8˚F. Mean bleeding times were 11.3, 9.7, 7.6, and 4.8 min; median 9.5, 7.8, 7, 5; and standard deviation of 5.6, 6, 3.7, 1.1 for room temperature platelets (Table 2).

**Table 2:** Mean bleeding time (min). min: Minutes

Platelets	Baseline	Pre-transfusion	Post-transfusion	One day post-transfusion	Three days post-transfusion	Seven days post-transfusion
4°C	4.8	15.9	12.7	12.4	9.5	6.3
22°C	4.5	14.6	11.3	9.7	7.6	4.8

Mean skin temperatures were 89.05, 88.39, 88.67, and 88.04˚F.

**Figure 1:**
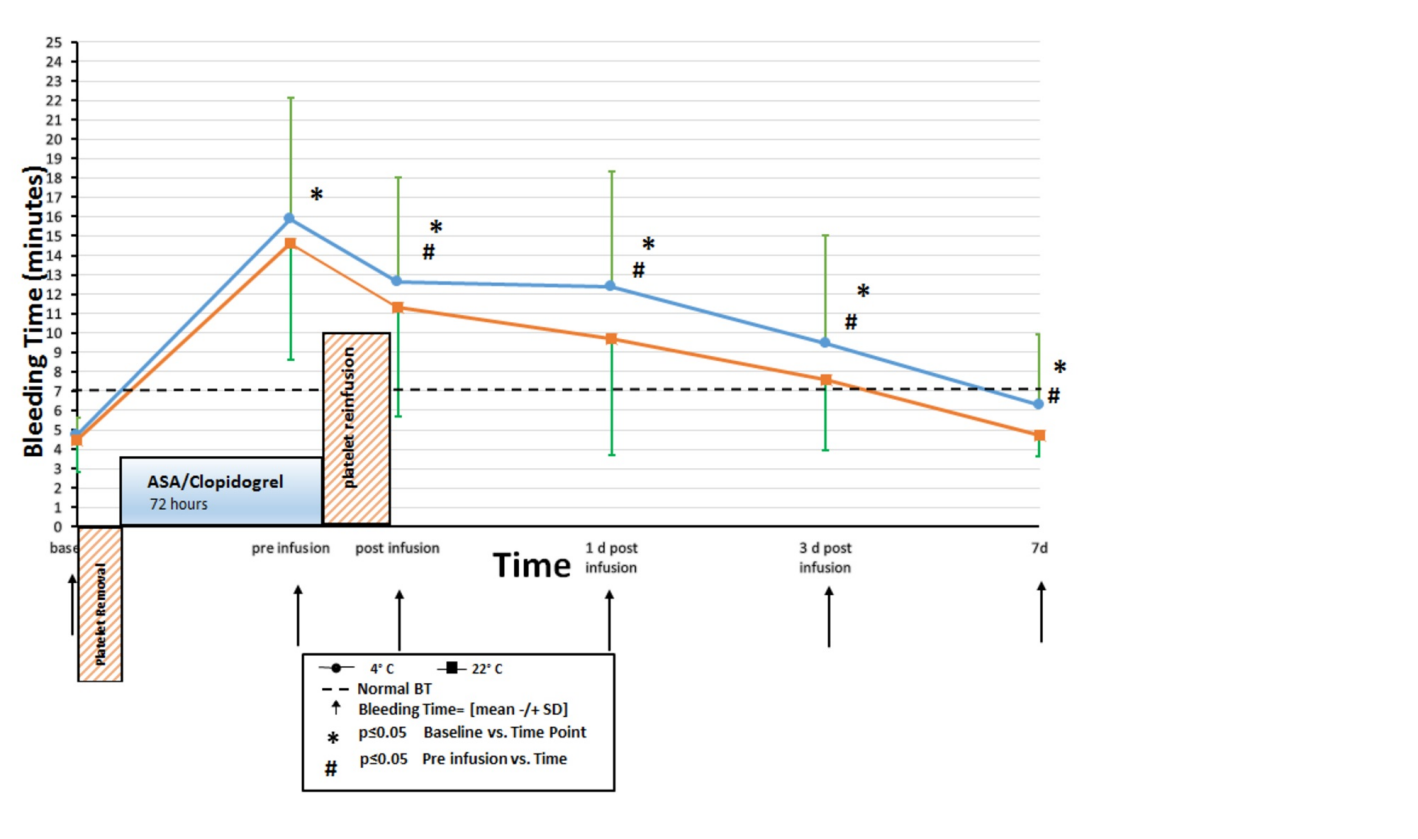
Study timeline.

Bleeding time decreased in both groups post-transfusion to slightly higher than baseline values in day seven. There were no statistically significant or clinically important differences between bleeding times in the 4˚C group and the room temperature platelet transfused group (Figure 1).

## Discussion

In this investigation, platelet infusion (irrespective of storage method) was not effective in restoring platelet function in subjects receiving aspirin and clopidogrel. Platelets are known to play a pivotal role in hemostasis after injury and there is a considerable amount of research suggesting that trauma significantly impairs platelet function [7-10]. For example, use of antiplatelet drugs in elderly patient with brain injuries has been associated with an increased mortality [11]. The likelihood of brain hemorrhage progression has been reported to rise five-fold in patients receiving antiplatelet therapy, with a doubling of both clinical deterioration and neurosurgical intervention [12].

Is there a value in transfusion of platelets to patients undergoing antiplatelet therapy?

Retrospective reviews focusing on the impact of platelet administration in reversing the deleterious effects of antiplatelet drugs are inconclusive [13]. Some studies show a significant improvement in trauma mortality with platelet infusion [14], while others showed a much worse outcome [11,15] or no impact [16]. In a prospective trial of elderly patients with traumatic intracranial hemorrhage, Joseph et al. [17] enrolled 28 patients suspected to be receiving high dose aspirin prior to admission. Twenty-two of the 28 patients had platelet dysfunction on admission by VerifyNow measurement. Unfortunately, 81% of patients with platelet hypofunction failed to improve with platelet infusion. Furthermore, there was no diminution of bleed progression or the need for neurosurgical intervention in those few patients who did improve platelet function with platelet administration.

Probably most concerning is the PATCH trial [18], where 41 centers across Europe randomized 190 patients taking antiplatelet therapy with non-traumatic intracranial bleeds were randomized, within six hours of imaging diagnosis, to standard therapy with or without platelet infusion. The group of patients receiving platelet transfusions had a significant increase in serious adverse effects and a doubling of the primary endpoint, death or dependence at three months (OR: 2.05, p = 0.0114).

What is the optimal test to assess platelet function?

The bleeding time (BT) has been used to assess hemostasis and remains the only in vivo test of platelet function [19]. Mielke et al. in 1969 demonstrated that the overall effect of aspirin is to prolong the bleeding time of a normal population. In this study, 60 normal males had a control bleeding time; they were given, in a double-blind fashion, either placebo or one gram of aspirin, and had a second bleeding time two hours later. The difference between the mean bleeding time after placebo and after aspirin was highly significant (p < 0.001) [20]. Subsequent clinical studies indicate a correlation between the volume of shed blood collected at the template BT site and the BT. The volume of shed blood correlates significantly with the BT in healthy persons and in patients with bleeding disorders, indicating that the BT test can predict excessive bleeding during and after surgery [19]. Another investigation in patients undergoing cardiopulmonary bypass (CPB) was designed to characterize the hematologic changes during and after CPB to assess the relationships among the hematologic measurements, the BT, and the postoperative nonsurgical blood loss. The reversal of the BT in the postoperative period was accompanied by significant increases in mean platelet volume and thromboxane B2 (TxB2) levels measured in the blood shed from the site of the BT determination. Postoperative BT correlated with the postoperative blood loss and established a direct relationship [19]. While bleeding time is an accurate, inexpensive and unique test of platelet function in vivo, it remains limited to experimental investigations due to high user variability.

The optimal in vitro assessment of platelet function was recently evaluated by Blais et al. [21]. They tested healthy individuals before and after eight to ten days of aspirin administration using five assays of platelet function. Measurement of platelet function by light transmission aggregometry with arachidonic acid and the VerifyNow system was found to be 100% sensitive and 96% specific for platelet dysfunction. These tests were much more accurate than thromboelastography (TEG) (sensitivity/specificity = 83%/76%), platelet count drop (sensitivity/specificity = 82%/87%), or urinary 11-dehydrothrombaxane B2 (sensitivity/specificity = 62%/82%). It would appear that VerifyNow may be the optimal in vitro test, as it is relatively inexpensive, fast and accurate compared to aggregometry.

What is the impact of storage temperature on restoration of platelet function?

Room temperature platelets were adopted by the U.S. blood industry over refrigeration after studies demonstrated greater platelet survival at 22°C [22]. This occurred despite excellent data which suggested that room temperature platelets have limited functional ability and fail to rapidly reverse the effects of aspirin when compared to fresh platelets [23]. In addition, these same investigators found that reversal of the antiplatelet effect required 12 units of platelets that were 48 hours old, compared to just four units with fresh platelets [23]. So, the decision to embrace room temperature platelets as the standard and solitary platelet product in the U.S. appears to have ignored the superior clotting efficacy of cold stored platelets [6]. A number of investigators have supported the notion that a platelet-containing blood product stored at 4°C becomes more hemostatic compared to those stored at 22°C [24,25]. One trial, performed in children, demonstrated significantly less postoperative bleeding after cardiac surgery in those patients who received refrigerated platelet-containing whole blood, compared with those who received reconstituted whole blood containing room temperature platelets [24]. There were no differences between the recipients of refrigerated whole-blood and fresh whole-blood, but platelet function, as measured with light impedance aggregometry, was poorest in samples from patients who received blood reconstituted with room temperature platelets [24]. In another trial in adults taking aspirin, bleeding times were significantly better in individuals infused with 4°C-platelets compared to those stored platelets at room temperature [25]. Findings from these randomized clinical trials confirm data from the 1970s that demonstrated the benefits of refrigerated platelets in more quickly correcting bleeding time when compared to room temperature platelets [26,27]. Reddoch et al. compared the effects of 22°C and 4°C storage on the functional and activation status of apheresis platelets. Apheresis platelets stored at 4°C maintained more viable metabolic characteristics, were hemostatically more effective, and released fewer proinflammatory mediators than apheresis platelets stored at RT over five days [28]. Nair et al. demonstrated that platelets stored at 4°C for up to five days were more responsive to aggregation stimuli and formed stronger clots presumably because of thicker fibrin strands, compared with platelets stored at room temperature. These data suggested that the superior functionality of cold-stored platelets might support faster hemostasis for acute bleeding in trauma patients compared with room temperature-stored platelets [29].

In light of these results and the importance of hemorrhage control in trauma patients, we performed a prospective observational study of platelet function in subjects taking aspirin and clopidogrel, before and after platelet transfusions. In our study, we determined that an autologous platelet transfusion had minimal effect on bleeding time in patients on aspirin and clopidogrel. Our analysis failed to identify any statistically significant differences in post-transfusion bleeding time when 4°C stored platelets were compared with platelets stored at room temperature. This data raises a fundamental question about the appropriate dosing of these blood products in acute patient care setting which was a limitation in our study. We only used one unit of platelets (equivalent to the old “six pack”, 3 x 10^11^ stored at either 22˚C or 4˚C for 72 hours).

Similar to our investigation, Pruller et al. [30] recently performed a study of healthy volunteers, who were administered two platelet packs (2 x 10^11^ platelets each) following three days of aspirin and clopidogrel. They performed in vitro measurement and found a variable response to platelet transfusions: light transmission aggregometry failed to improve until 24 hours post infusion, but P2Y12 receptor reactivity did recover from low platelet reactivity.

## Conclusions

In this randomized, single-blinded, crossover study of healthy volunteers on therapeutic doses of aspirin and clopidogrel, there was no difference in platelet function after autologous-platelet transfusion with units stored at room temperature or 4°C. We found that transfusion with platelets was ineffective in reversing the anti-platelet effects of aspirin and clopidogrel.
